# Character in Our Features

**Published:** 1898-01

**Authors:** A. O. Hunt

**Affiliations:** Chicago, Ill.


ARTICLE IV.

### CHARACTER IN OUR FEATURES.

BY A. O. HUNT, D. D. S., CHICAGO, ILL.

By close observation and careful records phrenology and physiognomy have been brought to such a state of perfection that by the outlines of the body, combined with its movements, it is possible to tell the character and disposition of an individual, as well as what those characteristics fit them for in the way of occupation. Palmistry also by the same careful observation and correct records can make some very close guesses as to those things which govern an individual, both past and present, by the lines, the hills and mountains as seen in the palm of the hand, in the shape and outlines of the fingers and thumbs.

Art and sculpture have by the same means been enabled to fix a classical standard of the ideal shape of the human form as seen in the external outlines on the surface of the body, exhibited by the position and action of the muscles. There has also been established a definite relation as to size in the circumference and length of parts of the body, as compared to each other.

The distance between the eyes, the length, width and position of the eyes, length of the nose, size of the mouth, etc., etc. And yet these features are so effected by their surrounding conditions that the human family is divided into five distinct races of people, and again for reasons into tribal and national characteristics, still retaining all the essentials of the human form. Wherever the study of the human form has been specialized both anatomy and physiology (form and function) have been well considered. It has engaged the best minds, and produced an extensive and valuable literature, such as "Darwin's Expression of Men and Animals," "Rimmer's Art Anatomy," various works on phrenology and palmistry, etc.—*Review*.

